# The Involvement of Long Non-Coding RNAs in Glioma: From Early Detection to Immunotherapy

**DOI:** 10.3389/fimmu.2022.897754

**Published:** 2022-05-10

**Authors:** Xiaoben Wu, Lei Yang, Jing Wang, Yingying Hao, Changyin Wang, Zhiming Lu

**Affiliations:** ^1^ Department of Clinical Laboratory, Shandong Provincial Hospital Affiliated to Shandong First Medical University, Jinan, China; ^2^ Department of Medical Engineering, Shandong Provincial Hospital Affiliated to Shandong First Medical University, Jinan, China

**Keywords:** glioma, biomarkers, long non-coding RNA, prognosis, immunotherapy

## Abstract

Glioma is a brain tumor that arises in the central nervous system and is categorized according to histology and molecular genetic characteristics. Long non-coding RNAs (lncRNAs) are RNAs longer than 200 nucleotides in length. They have been reported to influence significant events such as carcinogenesis, progression, and increased treatment resistance on glioma cells. Long non-coding RNAs promote cell proliferation, migration, epithelial-to-mesenchymal transition and invasion in glioma cells. Various significant advancements in transcriptomic profiling studies have enabled the identification of immune-related long non-coding RNAs as immune cell-specific gene expression regulators that mediates both stimulatory and suppressive immune responses, implying lncRNAs as potential candidates for improving immunotherapy efficacy against tumors and due to the lack of different diagnostic and treatments for glioma, lncRNAs are potential candidates to be used as future diagnostic, prognostic biomarker and treatment tools for glioma. This review’s primary purpose is to concentrate on the role of long non-coding RNAs in early glioma identification, treatment, and immunotherapy.

## Introduction

**Figure 1:**
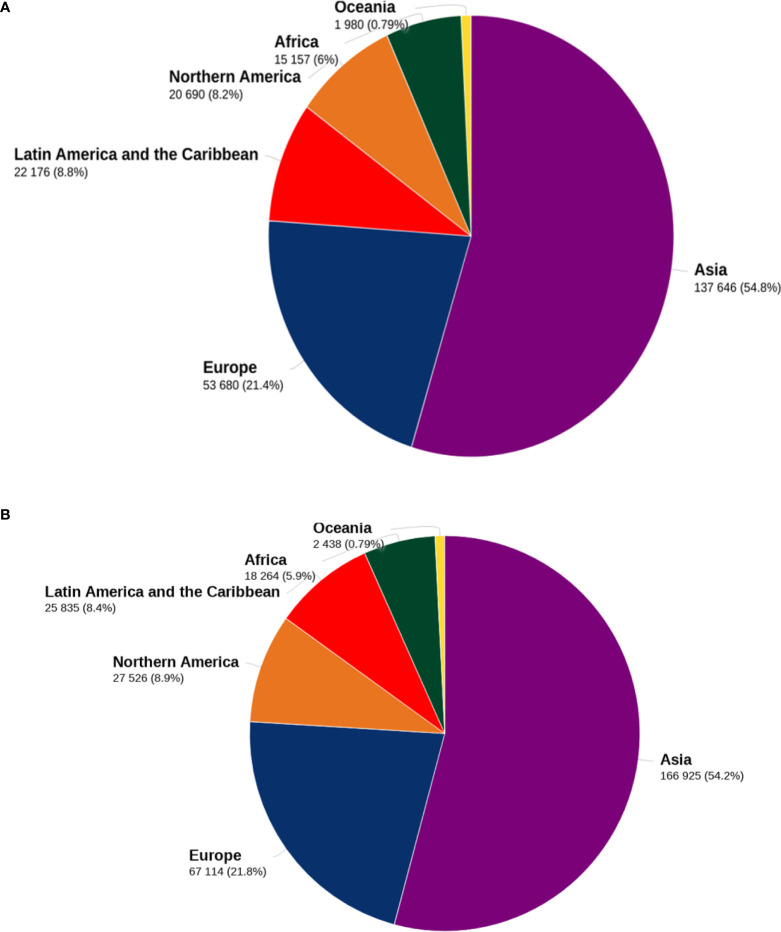
Death and incidence related to glioma. **Figure 1** shows the distribution of mortality **(A)** and incidence **(B)** of brain tumors for both sexes and all ages by 2020 in major geographical areas. The data sources and methodologies used for each global region are available online from the global health data exchange and GLOBALCAN 2020 at the Global Cancer Observatory.

Gliomas are the most often occurring malignant primary brain tumors in adults. They may occur anywhere in the central nervous system but are most often seen in the brain, developing in glial tissue (1). While these tumors are often malignant, some subtypes may not always behave malignantly. Moreover, they are the most frequently occurring primary intracranial tumor, accounting for 81% of malignant brain tumors (2). Although relatively rare, they cause significant mortality and morbidity (3). The United States’ incident rate is 3.20 per 100,000, and glioblastoma(GBM) occupies 60–70% of malignant glioma (4, 5). In addition, glioma is the 3rd most common cause of cancer mortality in people aged 15 to 34, accounting for 2.5 percent of cancer deaths globally; detailed data about mortality and incidences of glioma are present in **Figure 1**. Glioblastoma multiform accounts for 50% of gliomas, increasing frequency in persons over 65 (6, 7) and glioblastoma has a 5-year relative survival of ∼5% (8). Based on the world health organization, gliomas are classified based on the glial cells from which they originate, such as astrocytes, ependymal cells, and oligodendrocytes (9). Different risk factors have been identified to be related to glioma (10). Notably, they include an elevated risk associated with ionizing radiation exposure and lower risk associated with a history of allergies or atopic disorders (11).

Currently, standard glioblastoma(GBM) treatments include maximal surgical resection and combined radio-chemotherapy (12). Apart from GBM’s rapid proliferation, extensive invasion, intra- and intertemporal genetic heterogeneity, and treatment resistance, the poor prognosis of GBM patients is also due to a lack of insights into the molecular pathogenesis and an absence of timely and sensitive diagnostic and therapeutic monitoring tools (13). Therefore, it is crucial to elucidate molecular mechanisms underlying glioma development and progression and further explore reliable biomarkers.

Approximately 98% of RNA transcribed from human DNA is not translated into protein and thus, is called non-coding RNA (ncRNA) (14), and some of them are classified as long non-coding RNAs which interfere with the transcription and translation of genes without altering DNA sequences (15, 16). Long non-coding RNAs(lncRNAs) are longer than 200 nucleotides; dysfunctions of several lncRNAs have often been reported in different tumors; and these dysfunctional lncRNAs are associated with the pathogenesis and regulation of glioma’s cell proliferation, cell motility, angiogenesis, drug resistance, and radiation resistance (16, 17). Originally, non-coding portions of the genome were referred to as junk DNA due to their inability to be translated into proteins. In addition, long non-coding RNAs, whose transcripts are longer than 200 nucleotides, have already been engaged in many biological activities (18). LncRNAs are hypothesized to influence gene expression and epigenetic, transcriptional, and post-transcriptional modification, and their expressions are connected with several biological characteristics, including cell survival (19, 20). Long non-coding RNA dysregulation is increasingly being related to various human disorders. Notably, long non-coding RNAs have unique expression patterns across multiple tumor types and function as tumor suppressors or promoters.

Additionally, aberrant lncRNAs expression have been linked to the altered expression of genes involved in carcinogenesis, metastasis, and advancement of tumor stages in malignancies (21, 22). Rather than concentrating just on cancer cell proliferation and invasion, an emerging number of researches reveal the crucial function of the tumor microenvironment (TME) in the genesis and progression of gliomas. The TME is a complex structure composed of cancer cells and noncancerous cells such as endothelial cells, pericytes, fibroblasts, and immune cells (23). Microglia and macrophages make up to 30% to 50% of glioma cells, and tumor-associated microglia and macrophages (TAMs) in the brain are protumorigenic and increase proportionately to tumor grade (24, 25). Other immune cells, such as dendritic cells, have also been reported to play a critical role in cancer immunotherapy (26). As a result, it is essential to provide effective immunological predictors and prognostic markers to enhance glioma prognosis and guide specific treatment choices, including immunotherapy. This study aims to discuss the role of long non-coding RNAs in the early identification, treatment, and immunotherapy of glioma.

## Long Non-Coding RNAs

**Figure 2:**
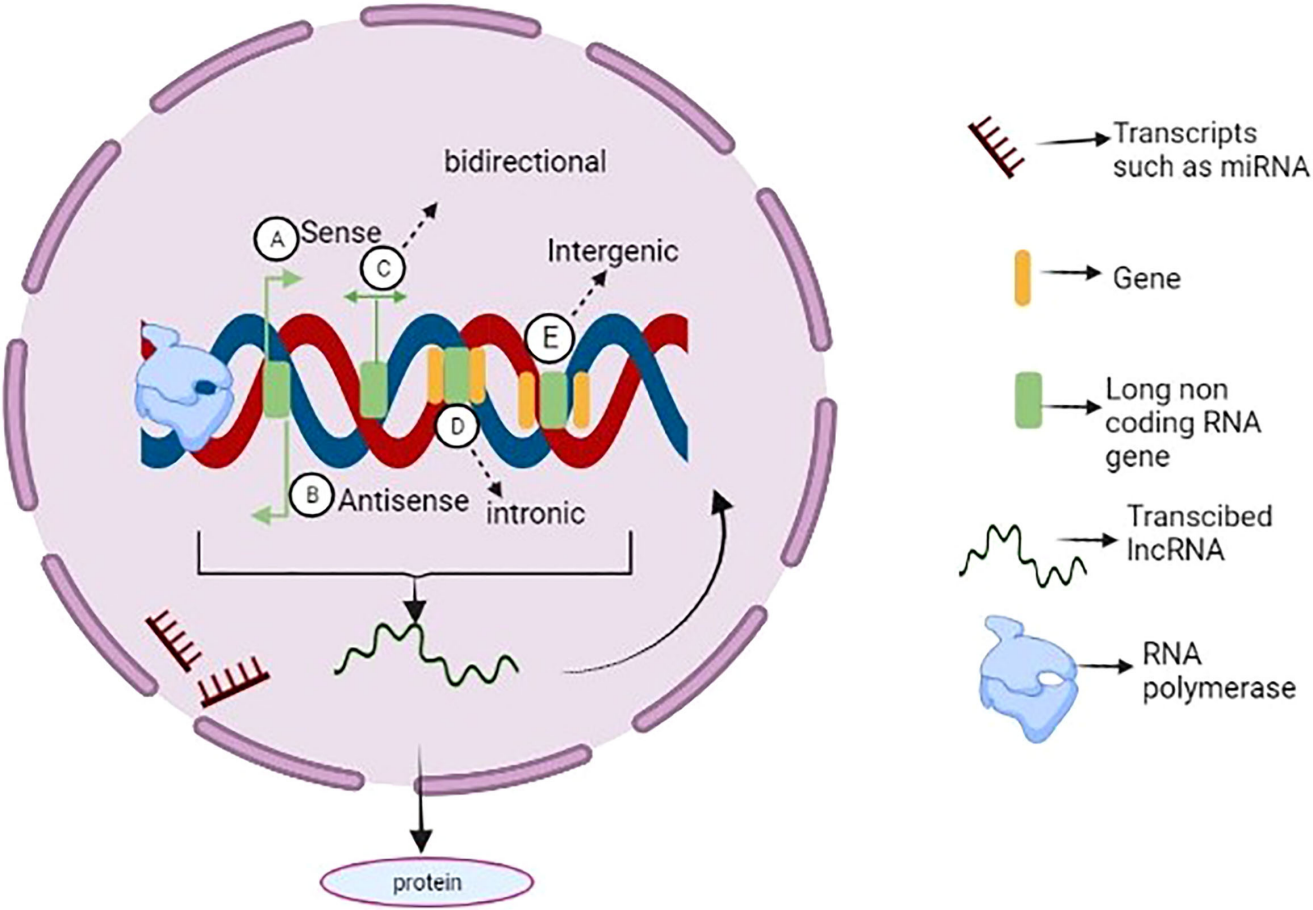
Long non coding RNA biogenesis and function. LncRNAs can be categorized into sense **(A)**, antisense **(B)**, bidirectional **(C)**, intronic **(D)**, and intergenic **(E)** lncRNAs based on their genomic location and Besides, some lncRNAs are reported to be transcribed from one of the strands of a DNA sequence. In addition to being transcribed by RNA polymerases, many of them are spliced, 5′ capped and polyadenylated. Although some lncRNAs can be translated into proteins, most of them serve as immediate regulators of genome function (e.g., microRNAs or very important binding partners of cellular components (e.g., proteins).

Long non-coding RNAs are non-coding RNAs longer than 200 nucleotides. During their biogenesisthey are systematically transcribed by RNA polymerase II, and long non-coding interfere with the transcription and translation of genes without altering DNA sequences. In addition, they are mostly polyadenylated and capped (27). Despite their increased nuclear distribution, more sparse structure, and lower evolutionary conservation, they have fewer exons than mRNAs (28). So, lncRNAs can be categorized into six categories based on their genomic positions: (a) sense, (b) antisense, (c) bidirectional, (d) intronic, (e)intergenic (**Figure 2**). Furthermore, lncRNA plays various roles in both the nucleus and cytoplasm. Their effects on the function and integrity of nuclear bodies play a role in chromatin remodeling, modifying chromosomal connections, transcription control, and post-transcriptional regulation of gene expression in the nucleus (29). Besides, they also regulate cellular mRNA turnover, translation, and post-translational modification within the cytoplasm. Micro-RNAs(mi-RNA) accessibility may be altered as competing endogenous RNAs (ceRNA) that act as a sponge for mi-RNAs.

Moreover, lncRNAs act as decoys for RNA binding proteins involved in the mRNA degradation process or promote protein binding to lncRNAs; subsequently, by altering messenger RNA or interacting with ribosomes, lncRNAs significantly influence translation (30). At last, lncRNAs participate in post-transcriptional modifications, such as phosphorylation and ubiquitination (31).

## Long Non-Coding RNA and Glioma Development

**Figure 3:**
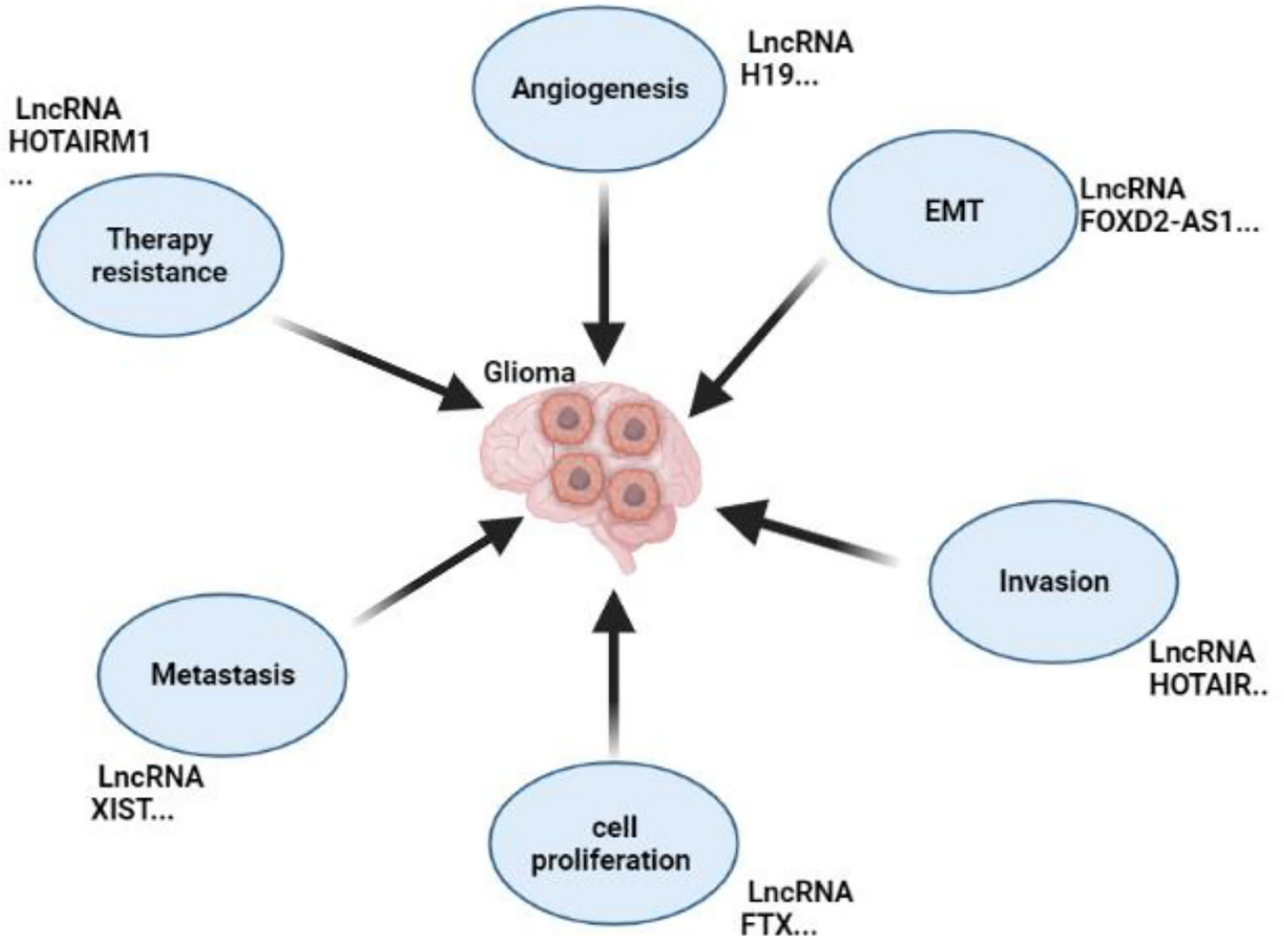
Roles of non-coding RNA in Glioma’s Development. This figure shows the roles of lncRNAs in glioma by promoting metastasis, EMT, invasion, proliferation, angiogenesis, and resistance to chemotherapy.

Numerous studies have shown that long non-coding RNAs play critical roles in glioma formation by stimulating critical processes (**Figure 3**). For instance, a study conducted with the primary goal of identifying long non-coding RNA and clarifying its function and mode of action in glioma formation showed that LPP Antisense RNA 2(LPP-AS2) promotes glioma carcinogenesis through a miR-7-5p/EGFR/PI3K/AKT/c-MYC feedback loop (32). Furthermore, a critical study discovered that clinical samples and cell lines had detectable quantities of FGD5 Antisense RNA 1(FGD5-AS1). By directly regulating the Wnt/-Catenin Pathway, the long non-coding RNA FGD5-AS1 promotes Glioma-related processes such as cell proliferation, migration, and invasion (33). Exosomes carrying long non-coding RNA have been reported to enhance glioma formation; for instance, recent research found that exosomes carrying Linc01060 increased glioma growth by effectively regulating the MZF1/c-Myc/HIF1 Axis (34); on the other hand, a study that examined the link between miR155HG and Annexin A2(ANXA2) to assess their malignancy in the formation of GBM discovered that the two, in combination with miR-185, enhanced glioma growth and development which proves the role of long non-coding RNA in glioma formation (35).

Furthermore, Zhang W et al. found that long non-coding RNA FTX was high in gliomas. By inhibiting miR-342-3p, this long non-coding RNA has been found to promote both cell proliferation and survival of glioma cells (36). Gliomas also undergo metastasis at this point in their development, and different researches have highlighted that long non-coding RNAs strongly influence and play a role in glioma metastasis. For example, Huixiao Dong et al. demonstrated that camp responsive element binding protein 1(CREB1) triggers long non-coding adjacent opposite strand RNA 1 (FOXD2-AS1), leading to increased cell proliferation and metastasis in gliomas *via* miR-185 sponging by targeting AKT Serine/Threonine Kinase 1(AKT1) (37). Besides, Chixing Luo and his colleagues found a correlation between miR-133a/SOX4 and lncRNA XIST promoting glioma proliferation and metastasis (38).

There are shreds of evidence showing that long non-coding RNAs may be involved in the promotion of glioma’s angiogenesis, which is a very ultimate factor that affects glioma’s development; recently, a study reported that lncRNA H19 promotes angiogenesis by activating the miR-138/HIF-1*/VEGF pathway, suggesting that targeting this pathway could be essential for successfully stopping angiogenesis (16), also, a study conducted by Zhihua Cheng et al., reported that long non-coding RNA XIST inhibited miR-429, led to glioma tumorigenesis and angiogenesis (39). Furthermore, it has also been reported that long non-coding RNAs promote EMT, an important event in cancer progression, including gliomas. For instance, research conducted by Juan Zhao showed that lncRNA FOXD2-AS1 speeds up numerous crucial events in gliomas, such as proliferation, metastasis, and EMT (40). It is evident that long non-coding RNAs play an essential role in the development of glioma by promoting different important glioma events. Additional information can be found in **Table 1**.

**Table 1:** Long Non-Coding RNA involved in glioma development.

lncRNA	Involved MicroRNA	Outcome	References
**HOTAIRM1**	miR-495-3p and miR-129-5p	Promoting cell proliferation, EMT and TMZ resistance	(41)
**lncRNA oncogene NEAT1**	miR-132	promoting glioma development	(42)
**RPL34-AS1**	VEGFA	promotes proliferation and angiogenesis	(43)
**lncRNA TMPO-AS1**	miR-383-5p	Promotes Proliferation and Invasion	(44)
**Longnon-coding RNA LINC00958**	miR-203/CDK2	Promoting cell proliferation, invasion and migration and inhibiting apoptosis	(45)
**Long non-coding RNA FGD5-AS1**	wnt/β-Catenin	Glioma Cell Proliferation, Migration and Invasion	(33)
**Long non-coding RNA OIP5-AS1**	miR-410	glioma progression	(46)

## Long Non-Coding RNAs and Glioma Tumor Immune Microenvironment

Long non-coding RNAs are adaptable molecules that interact with RNA, DNA, and proteins to influence the expression of protein-coding genes (47). Several immune cells contain lncRNAs that affect both innate and adaptive immunity. In recent years, researchers have become more aware of the importance of lncRNAs in controlling the tumor immune microenvironment (TME) (48, 49). Furthermore, tumor microenvironments contain immunosuppressive cells that inhibit antitumor immunity (50). Patients with malignant glioma present several immunological defects, including CD4 lymphopenia, elevated regulatory T cells (Tregs) in peripheral blood, immune-suppressive macrophage infiltration, and decreased Th1 cytokine production impaired cell-mediated immunity (51, 52). Besides, some studies have examined lncRNA expression patterns in glioblastoma and have discovered immune-related expression patterns (53, 54), with proven predictive value in glioma development and prognosis.

Moreover, lncRNAs control inflammation genes, which may affect the immune system. The lncRNA nuclear paraspeckle assembly transcript 1 (NEAT1), for example, modulates the transcription of IL-8 and affects the activity of various immune cells (55); this is the same as lncRNA-Cox2, which has been found to inhibit the SWI/SNF complex’s chromatin remodeling activity in macrophages in addition to suppressing inflammatory genes in macrophages (56); and, another study found that hypoxia-related lncRNA in lower-grade gliomas correlates with prognosis and regulates the immune microenvironment (57). Understanding immune-related lncRNAs in gliomas may help understand the involvement of lncRNA in TME and the immune system which will significantly help to get more ways to deal with glioma aggressiveness and development.

## Clinically Leading Roles of Long Non-Coding RNA in Glioma

### Long Non-Coding RNAs as Diagnostic and Prognostic Biomarkers for Glioma

It is essential to identify cancer as early as possible to fight it in its early stage. Identifying different types of cancer, including gliomas, requires biomarkers (58, 59). Upregulation and downregulation of long non-coding RNAs can reveal other cancer statuses, making them suitable biomarkers for specific forms of cancer, such as gliomas (60). Numerous studies demonstrate that lncRNAs have thus become a valuable tool for predicting gliomas. For example, researchers have developed a nomogram (based on m6A-LPS, age, and WHO grade) that is a good predictor of low-grade gliomas (LGGs) patients’ overall survival in both data sets, concluding that N6-Methylandenosine-Related lncRNAs are particularly useful biomarkers for predicting overall survival in lower-grade gliomas (61). In addition, a different study found that serum levels of lncRNA HOX antisense intergenic RNA (**HOTAIR**) were elevated in tumor samples compared with standard specimens. In addition, they found that long non-coding RNA HOTAIR was highly diagnostic, suggesting that it could serve as a potential biomarker to diagnose and predict gliomas (62).

According to a study, which also examined the expression of MAGI2 antisense RNA 3(MAGI2AS3) and its clinical significance in gliomas, it was found that the expression of the lncRNA was higher in tumors than in normal samples; in glioma patients, there was a direct correlation between the MAGI2-AS3 expression and cancer world health organization (WHO) grade and The Karnofsky Performance Scale (KPS) score. Kaplan-Meier analysis \showed that patients with low MAGI2 antisense RNA 3 (MAGI2-AS3) levels had a worse overall survival rate than those with high MAGI2-AS3 levels. Furthermore, the researchers discovered that the expression of MAGI2-AS3 in glioma tissues was an independent predictor of overall survival when analyzed by multivariate logistics (63).

Moreover, Jun-Chi Mei et al. revealed that the amount of long non-coding RNA ELF3 antisense RNA 1(ELF3-AS1) was significantly more tremendous in glioma specimens than in non- tumor samples adjacent to the tumor. Considering its prognostic and diagnostic importance, it could be regarded as a viable marker of glioma (64).On the other hand, F Shang and his Colleagues found that PXN-AS1-L was overexpressed in glioma. It can be used as a potential biomarker for glioma patients’ unfavorable outcomes (65). There’s been a link between metastasis-related lung adenocarcinoma transcript 1 (MALAT1) expression and greater chemoresistance to TMZ, suggesting it could be a prognostic marker for glioma (66). According to a recent analysis of LGG data from the cancer genome atlas (TCGA), 16 immune-related lncRNAs were significantly linked to patient outcomes (67).

Additionally, six lncRNA signatures associated with immunity within the TCGA offer excellent prognostic information for patients with GBM (68). Also, Wang S and his colleague revealed that the long non-coding RNA RPSAP52 could help predict postoperative survival for people with GBM since it is expressed in these cells (69). It was also reported that ADAMTS9-AS2 and HOXA11-AS gene expression increase with tumor grade. In addition, the CASC2 gene expression decreased dramatically with rising tumor grade, and the authors concluded that lncRNA ADAMTS9-AS2, HOXA11-AS, and CASC2 are potential biomarkers for glioma prognosis (70). Continuing to study lncRNAs will provide more effective and sensitive biomarkers to diagnose glioma early.

### LncRNAs in Glioma Treatment

#### Treatment Agents for Glioma

Due to the diversity of lncRNA activity, these transcripts are attractive as potential therapeutic targets due to their low abundance; broad and severe phenotypic effects (71). There is a poor prognosis for patients with GBM due to the heterogeneity and aggressive nature of glioma cells, an immune-privileged brain environment, the absence of effective treatments, and the inability to penetrate the BBB of most therapies (72). Another significant study developed a generalizable approach for rapidly identifying novel therapeutic targets using CRISPRi-based genome-wide screening. Additionally, this research discovered that antisense oligonucleotides targeting lncGRS-1 suppressed glioma tumor formation in 3D culture and increased the radiation sensitivity of glioma cells (73). According to Ke Zhou et al., nuclear enriched abundant transcript 1 (NEAT1) silencing inhibits motility and invasion of glioma cells by modifying SRY-Box Transcription Factor 2 (SOX2), which miR-132 targets. These findings establish NEAT1 as a potential therapeutic target (42). Earlier this year, it was discovered that inhibiting Wnt/-catenin signaling suppresses the growth of glioblastoma cells by blocking the processing of the lncRNA MIR22HG; for people with glioblastoma, targeting this RNA might represent a novel therapeutic strategy (74).

Additionally, Ning Guan and colleagues noticed that lncRNA Neuroblastoma Associated Transcript 1(NBAT1) was able to halt the evolution of glioma through the miR-21/SOX7 axis, demonstrating its therapeutic potential (75). Another challenge that physicians face while treating a patient with glioma is treatment resistance. LncRNAs have been implicated in improving resistance to several medicines. For instance, lncSBF2-AS1 SBF2-AS1 expression was elevated in TMZ-resistant glioblastoma (GBM) cells and tissues. That overexpression of SBF2-AS1 facilitated TMZ resistance but silenced SBF2-AS1 sensitized resistant GBM cells to TMZ (76). Furthermore, another research revealed that directly targeting miR-10a, the lncRNA TUSC7 decreased temozolomide resistance in glioblastoma (77). Yu L et al. demonstrated that silencing Long intergenic non-protein coding RNA 00475 could act as a tumor suppressor in gliomas *via* microRNA-449b-5p-dependent AGAP2 upregulation; this indicates the potential for its therapeutic use in the treatment of gliomas (78).

Moreover, another research discovered that lncRNA brain cytoplasmic RNA 1(BCYRN1) served as a miR-619-5p sponge to regulate the cue domain containing 2(CUEDC2) expression in the PTEN/AKT/p21 pathway; this combination reduced glioma development. This research indicated that the lncRNA brain cytoplasmic RNA 1(BCYRN1)acts as a tumor suppressor and may help diagnose and treat glioma (79). The studies discussed above provide critical evidence that lncRNAs may be used as therapeutic agents by suppressing glioma.

#### LncRNAs as Treatment Response Indicators

Determining therapy responses is critical throughout the cancer treatment process. LncRNAs are among the most effective techniques for monitoring glioma therapy. For example, metastasis-related lung adenocarcinoma transcript 1(MALAT1) has a role in tumor chemosensitivity and is overexpressed in temozolomide (TMZ)-resistant glioblastoma cells. Its silencing lowered TMZ resistance *in vivo* and *in vitro* (80). Similarly, TMZ-resistant tumors displayed reduced maternally expressed 3(MEG3) expression (41). Cisplatin sensitivity was boosted by increasing MEG3 expression. At the same time, chemoresistance was created by silencing MEG3 using si-RNA (81), and the authors discovered that MEG3 is a critical tool for tracking chemotherapy response in glioblastoma. Additionally, it was reported that SHG-44 cells resistant to paclitaxel expressed high levels of plasmacytoma variant translocation 1(PVT1) *in vitro* and the knocking down of this gene improved the chemo-response; the above effects resulted in this long coding RNA being able to track the response to paclitaxel in glioma patients.

### LncRNA Are Crucial Players in Glioma Immunotherapy

The lncRNAs are crucial in many immune responses, including inflammation, cell differentiation, immune cell maturation, and infiltration (82, 83). The expression of immune-related lncRNAs can also be used to classify tumors since their expression is correlated with immunologic molecules such as cytokines and chemokines (84, 85). Due to these circumstances, it is vital to identify immune-related lncRNAs that affects glioma-associated immune cells, which can help develop glioma immunotherapies. For example, one study found a significant connection between immune-related lncRNA levels and immune cell infiltration in various tumors. This research comprehensively charted the landscape of lncRNA regulation in the immunome across 33 cancer types, showing that cancers with similar tissue origin are likely to share lncRNA immune regulators. Moreover, the immune-related lncRNAs are likely to show expression perturbation in cancer and lncRNAs are significantly correlated with immune cell infiltration. They found that immune-related lncRNAs can help prioritize cancer-related lncRNAs and further identify three molecular subtypes (proliferative, intermediate, and immunological, makinge them potential oncogenic biomarkers (86).

In response to toll-like receptors (TLRs) activation, NF-κB (Nuclear factor-κB) induces or suppresses the expression of lncRNAs in various immune cells, which influences immune responses (87). Specific lncRNAs have been linked to tumor growth by dysregulation of proliferation, apoptosis, metastasis, and angiogenesis. Furthermore, lncRNAs mainly target immunological checkpoints and cytokines, facilitating the establishment of an immunosuppressive milieu conducive to tumor proliferation and medication resistance. For instance, a study of the cancer genome atlas (TCGA) database indicated that lncRNA AC003092.1 is associated with an immunosuppressive microenvironment in glioblastoma (88). Through the research of the TCGA and the Chinese glioma genome atlas (CGGA) databases, researchers discovered that lncRNA SBF2-AS1 is linked with immunity in lower-grade glioma (89).

Additionally, researchers have revealed that the expression of the lncRNA MIR155 host gene (MIR155HG) is elevated in GBM tissues in comparison to their equivalent standard counterparts and that its expression is associated with immune checkpoint inhibitors such as Programmed cell death one ligand (PD-L1) and T cell immunoglobulin and mucin domain 3 (TIM-3) in GBM and that its expression is associated with poor prognosis (90). It was also found that the lncRNA RP11-838N2.4 enhanced the cytotoxic effects of temozolomide in glioblastoma cells by inhibiting miR-10a functions (91). Furthermore, another study identified nine immune-associated lncRNAs predictive of low-grade glioma (AC009283.1, AC009227.1, AL121899.1, LINC00174, LINC02166, AC018647.1, AC061961.1, NRAV, and LINC00320). They concluded that these nine lncRNAs represent independent prognostic factors for developing low-grade gliomas and that these RNAs modulate immunological responses and cancer pathways to affect tumorigenesis and prognosis of patients with low-grade gliomas (92). Functional analysis of immune-related lncRNAs revealed that the differences in overall survival between groups with high and low RS might be explained by differences in cell differentiation, microtubule polymerization, and other processes. This study demonstrated that those immune-related lncRNAs contribute to glioma pathogenesis and clinical treatment, and It may be a potential therapeutic for glioma diagnosis (53).

Furthermore, another research discovered 11 immunological lncRNAs that may be used as prognostic indicators for individuals with LGG. The prognosis prediction performance of the nomogram produced according to these lncRNAs is quite effective. In addition, these 11 immune lncRNAs are associated with the infiltration of immune cell subtypes in tumor tissues, which have a possible role in the development of LGG *via* several routes (93). Furthermore, in glioblastomas, a research identified four prognostic lncRNAs (AK098425, AL833059, AK056155, and CR613436) that co-express protein-coding genes and are strongly associated with immune responses, including the inflammatory response, the innate immune response, and B cell-mediated immunity (68). The lncRNA HOTAIR myeloid-specific 1 (HOTAIRM1), which is overexpressed in gliomas, promotes malignancy by interacting with miR-129-5p as a ceRNA associated with immune activation, which enhances T cell-mediated immune responses (94).. Therefore, these lncRNAs are advantageous for developing immunotherapies specific to glioma. Considering the studies mentioned above, it is evident that studying in deep long non-coding RNAs will reveal new immunotherapeutic targets to develop various treatment options for cancer. This will include effective treatments for gliomas, as well.

### Clinical Trials Related to lncRNAs and Immunotherapies in Glioma Diagnosis and Treatment

Clinical trials assist scientists and physicians in evaluating diagnostic and therapeutic methods for different types of cancer, including gliomas. Numerous clinical studies have been conducted to demonstrate the use of lncRNA in treating and diagnosing a variety of malignancies, including hepatocellular carcinoma (NCT05088811), lung cancer, and prostate cancer (NCT03830619). Additionally, some other clinical trials on immunotherapies for glioma have been conducted, such as a phase I clinical trial evaluating cellular immunotherapy with intratumoral alloreactivity cytotoxic T lymphocytes and interleukin-2 for the treatment of recurrent malignant gliomas or meningioma one of such clinical studies (NCT01144247). However, no clinical studies investigated lncRNAs as diagnostic or therapeutic tools for glioma. Thus, there is a critical need for more clinical studies to demonstrate the functions of lncRNA in glioblastoma and establish them as standard therapy and biomarkers for glioma and other malignancies; details on the aforementioned clinical trials and others are in **Table 2**.

**Table 2:** Examples of clinical trials done on the roles of lncRNAs as biomarkers or in treatment agents.

Status of the clinical trials	Objectives	Condition of the disease	Clinical trial Identifier
**Completed**	Study the sensitivity and specificity of serum exosome non-coding RNA as a lung cancer biomarker	Lung Cancer	NCT03830619
**Recruiting**	Researchers examined the relationship between lncRNA H19 and Insulin-Like Growth Factor 1 Receptor (IGF-1R) mRNA gene expressions in lymph nodes from HCC and T2DM patients to test whether there may be a pathophysiological link between these two diseases that may become a therapeutic target for both.	Hepatocellular Carcinoma Type 2 Diabetes Cancer	NCT04767750
**Not yet Recruiting**	Evaluate the clinical utility of detecting lncRNA CCAT1 expression in the diagnosis of colorectal cancer patients & its relation to tumor staging	Glioma, Glioblastoma Multiforme	NCT04269746
**Recruiting**	Determining the Role of LncRNAs WRAP53 and UCA-1 as Potential Biomarkers in Diagnosis of Hepatocellular Carcinoma	Hepatocellular Carcinoma, Liver, Cirrhosis, Diagnoses Disease.	NCT05088811

## Conclusions

Glioblastoma is one of the fatal types of brain cancer. While surgical and radiation advancements have been beneficial, the development of non-invasive treatments remains a necessity for genuinely outstanding treatment alternatives. Long non-coding RNAs have recently received renewed attention in glioma research and may be a viable tool for targeted therapy. It is well established that lncRNAs engage in various cellular regulatory mechanisms that influence the development of carcinogenesis. Numerous aberrant lncRNAs have been linked with glioma. They have been implicated in almost all of its characteristics, including cell proliferation, motility, angiogenesis, stemless, tumor recurrence, severity, and chemoresistance. Innovative biochemical and molecular techniques have aided in elucidating how lncRNAs govern and regulate many functionalities. While it is encouraging to discover potential diagnostics and therapies to increase glioma survival utilizing lncRNAs, most lncRNAs have different undefined properties. The distribution of medications into the brain across an intact BBB is an essential element to consider when developing helpful therapeutics. Due to their low stability and poor drug absorption, therapies based on lncRNAs are limited to *in vivo* usage. Thus, future research should focus on detecting the cell-to-cell heterogeneity of lncRNAs in gliomas, as this would aid in the creation of innovative RNA-based methodologies for treating this malignancy and instill fresh confidence in patients with glioma also, as recent research has shown that lncRNAs operate as epigenetic regulators that promote innate immune memory responses. Further investigation of immune-related lncRNA in terms of immune response prediction is necessary, as they may serve as candidates for particular immune cell-mediated tumor evasion and cellular death.

## Author Contributions

XW designed and drafted the manuscript. LY, JW, YH, CW, and ZL discussed and revised the manuscript. All authors read and approved the final manuscript.

## Funding

This study was supported by the Clinical Medicine Science and Technology Innovation Project of JINAN (No. 202019020).

## Conflict of Interest

The authors declare that the research was conducted in the absence of any commercial or financial relationships that could be construed as a potential conflict of interest.

## Publisher’s Note

All claims expressed in this article are solely those of the authors and do not necessarily represent those of their affiliated organizations, or those of the publisher, the editors and the reviewers. Any product that may be evaluated in this article, or claim that may be made by its manufacturer, is not guaranteed or endorsed by the publisher.

## Abbreviations

NEAT1, Nuclear enriched abundant transcript 1; Cox-2, Cyclooxygenase-2; FGD5-AS1, FGD5 antisense RNA 1; MZF1, Myeloid zinc finger 1; HIF1, Hypoxia-inducible factor 1-alpha; ANXA2, Annexin A2; CREB1, CAMP Responsive element-binding protein 1; FOXD2-AS1, Forkhead box D2 adjacent apposite strand RNA 1; XIST, X-inactive specific transcript;

VEGF, Vascular endothelial growth factor; EMT, Epithelial-to-Mesenchymal Transition;

HOTAIRM1, HOXA transcript antisense RNA, myeloid-Specific 1; TMPO-AS1, TMPO antisense RNA 1; TME, tumor microenvironment;

LGGs, Low-grade gliomas; MAGI2-AS3, MAGI2 antisense RNA 3; ELF3-AS1, ELF3 antisense RNA 1; MALAT1, Metastasis-associated lung adenocarcinoma transcript 1;

TMZ, Temozolomide; AGAP2, ArfGAP with GTPase domain, ankyrin repeat, and ph domain 2; BCYRN1, Brain cytoplasmic RNA 1; CUEDC2, CUE domain containing 2; BBB, Blood-brain barrier.
